# A crucial role for tumor necrosis factor receptor 1 in synovial lining cells and the reticuloendothelial system in mediating experimental arthritis

**DOI:** 10.1186/ar2974

**Published:** 2010-04-06

**Authors:** Onno J Arntz, Jeroen Geurts, Sharon Veenbergen, Miranda B Bennink, Ben T van den Brand, Shahla Abdollahi-Roodsaz, Wim B van den Berg, Fons A van de Loo

**Affiliations:** 1 Rheumatology Research and Advanced Therapeutics, Department of Rheumatology, Radboud University Nijmegen Medical Centre, 6525 GA Nijmegen, The Netherlands

## Abstract

**Introduction:**

Rheumatoid arthritis (RA) is an autoimmune inflammatory disease that mainly affects synovial joints. Biologics directed against tumor-necrosis-factor (TNF)-α are efficacious in the treatment of RA. However, the role of TNF receptor-1 (TNFR1) in mediating the TNFα effects in RA has not been elucidated and conflicting data exist in experimental arthritis models. The objective is to investigate the role of TNFR1 in the synovial lining cells (SLC) and the reticuloendothelial system (RES) during experimental arthritis.

**Methods:**

Third generation of adenovirus serotype 5 were either injected locally in the knee joint cavity or systemically by intravenous injection into the retro-orbital venous sinus to specifically target SLC and RES, respectively. Transduction of organs was detected by immunohistochemistry of the eGFP transgene. An adenoviral vector containing a short hairpin (sh) RNA directed against TNFR1 (HpTNFR1) was constructed and functionally evaluated *in vitro *using a nuclear factor-kappaB (NF-κB) reporter assay and *in vivo *in streptococcal cell wall-induced arthritis (SCW) and collagen-induced arthritis (CIA). Adenoviruses were administered before onset of CIA, and the effect of TNFR1 targeting on the clinical development of arthritis, histology, quantitative polymerase chain reaction (qPCR), cytokine analyses and T-cell assays was evaluated.

**Results:**

Systemic delivery of Ad5.CMV-eGFP predominantly transduced the RES in liver and spleen. Local delivery transduced the synovium and not the RES in liver, spleen and draining lymph nodes. *In vitro*, HpTNFR1 reduced the TNFR1 mRNA expression by three-fold resulting in a 70% reduction of TNFα-induced NF-κB activation. Local treatment with HpTNFR1 markedly reduced mRNA and protein levels of interleukin (IL)-1β and IL-6 in SLC during SCW arthritis and ameliorated CIA. Systemic targeting of TNFR1 in RES of liver and spleen by systemic delivery of Ad5 virus encoding for a small hairpin RNA against TNFR1 markedly ameliorated CIA and simultaneously reduced the mRNA expression of IL-1β, IL-6 and Saa1 (75%), in the liver and that of Th1/2/17-specific transcription factors T-bet, GATA-3 and RORγT in the spleen. Flow cytometry confirmed that HpTNFR1 reduced the numbers of interferon (IFN)γ (Th1)-, IL-4 (Th2)- and IL-17 (Th17)-producing cells in spleen.

**Conclusions:**

TNFR1-mediated signaling in both synovial lining cells and the reticuloendothelial system independently played a major pro-inflammatory and immunoregulatory role in the development of experimental arthritis.

## Introduction

Rheumatoid arthritis (RA) is a chronic and systemic autoimmune disease that mainly affects synovial joints and is characterized by inflammatory synovitis, ultimately leading to the destruction of cartilage and bone. The central role for tumor necrosis factor-alpha (TNFα) in RA pathogenesis has been extensively demonstrated in experimental arthritis by successful treatment of murine collagen-induced arthritis (CIA) with TNFα antibodies [1,2] and development of arthritis in transgenic mice overexpressing human TNF [3]. Most importantly, TNFα has been identified as a key cytokine in human RA [4], which has led to the development of effective treatment of disease by administration of neutralizing TNF antibodies [5,6].

TNFα signaling is mediated via two distinct receptors encoded by the genes *Tnfrsf1a *(TNFR1) and *Tnfrsf1b *(TNFR2). The TNF receptors are transmembrane glycoproteins and share only 28% homology, predominantly between their extracellular domains. Both TNFR1 and TNFR2 activate a wide range of proinflammatory signal pathways, leading to activation of nuclear factor-kappa-B (NF-κB) and c-Jun N-terminal kinase, via recruitment of TNF receptor-associating factors (reviewed in [7]). Attenuation of CIA in TNFR1-deficient mice has demonstrated a dominant role of this receptor in disease [8,9]. Recent investigations on the cell-specific contribution of TNFR1-mediated signaling in RA pathogenesis have revealed remarkably different functions of TNFR1 in mesenchymal or hematopoietic compartments. Cells from the prior compartment - in particular, synovial fibroblasts (SFs) - have been identified as the primary targets for TNFα in the development of arthritis [10]. In contrast, TNFR1-mediated signaling in cells from the latter compartment, such as leukocytes, exerts an anti-inflammatory function [11,12].

This cell specificity of TNFR1 function is highly relevant to the safety and efficacy of treatments that target TNFα signaling. Scintigraphic imaging of the biodistribution of radiolabeled anti-TNF after systemic administration in RA patients has shown that antibodies accumulate not only in inflamed joints but also in the liver and spleen [13]. However, the function of TNFR1 expression in these secondary lymphoid organs and its contribution to RA pathogenesis remain to be elucidated.

In this study, we investigated the effects of TNFR1-mediated signaling in synovial lining cells (SLCs) and the reticuloendothelial system (RES) during experimental arthritis. To this end, we used cell-specific RNA interference (RNAi)-mediated silencing of TNFR1 based on adenoviral delivery of a short hairpin RNA (shRNA)-expressing construct.

## Materials and methods

### Animals

Male 10- to 12-week-old DBA/1J and C57BL/6 mice were obtained from Janvier (Janvier, Elavage, France). During viral experiments, mice were housed in HEPA-filtered individually ventilated cages. The animals were fed a standard diet with food and water *ad libitum*. All *in vivo *studies complied with national legislation and were approved by the local authorities on the care and use of animals.

### Induction of collagen-induced arthritis

Bovine collagen type II (bCII) was dissolved in 0.05 M acetic acid to a concentration of 2 mg/mL and was emulsified in equal volumes of Freund's complete adjuvant (2 mg/mL of *Mycobacterium tuberculosis *strain H37Ra; Difco Laboratories, now part of Becton Dickinson and Company, Franklin Lakes, NJ, USA). DBA1/J mice were immunized intradermally at the base of the tail with 100 μL of emulsion (100 μg of bCII). On day 21, the mice were given an intraperitoneal booster injection of 100 μg of bCII dissolved in phosphate-buffered saline (PBS). Mice were killed on day 31 by cervical dislocation.

### Streptococcal cell wall (SCW) preparation and induction of SCW arthritis

*Streptococcus pyogenes *T12 organisms were cultured overnight in Todd-Hewitt broth. Cell walls were prepared as described previously [14]. The resulting supernatant obtained after centrifugation at 10,000 *g *contained 11% muramic acid. Unilateral arthritis was induced by intra-articular (i.a.) injection of 5 μg of streptococcal cell wall (SCW) fragments (rhamnose content) in 6 μL of PBS.

### Cell culture

Mouse embryonic fibroblasts (NIH 3T3) stably transfected with a 5 × NF-κB-luciferase reporter were cultivated in Dulbecco's modified Eagle's medium (DMEM) supplemented with 1 mM pyruvate, penicillin-streptomycin (Lonza, Basel, Switzerland), and 5% fetal calf serum (FCS). Cells were kept at 37°C in a humid atmosphere containing 5% CO_2_.

### Plasmids

For cloning, we used *Pfu *DNA polymerase (Stratagene, La Jolla, CA, USA) and T4 DNA Ligase (New England Biolabs, Inc., Ipswich, MA, USA). All generated constructs were verified by sequencing. The U6 promoter was polymerase chain reaction (PCR)-cloned from mouse genomic DNA into *XbaI/SalI *sites of pShuttle (kind gift of Bert Vogelstein, Howard Hughes Medical Institute, Baltimore, MD, USA) to give pShuttle-U6 using primers forward 5'-TCTAGAGATCCGACGCCGCCATCTCTA-3' and reverse 5'-GTCGACGTTAACAAGGCTTTTCTCCA-3'. The target sequence for silencing the Tnfrsf1a gene [EMBL:M60468] was ATCTTCGGTCCTAGTAACT (base pairs 1095 to 1113), and we used ACTCATGTCTTGATCAGCT (no complementary sequence in murine genome) as scrambled control sequence. The silencing cassette was constructed using the following oligonucleotides: forward 5'-TG-*target*-TTCAAGAGA-*target reverse complimentary*-TTTT-TGCA-3' and reverse 5'-AAAA-*target*-TCTCTTGAA-*target reverse complimentary*-CA-3', where the loop and polyA sequences are underlined and bold, respectively. Oligonucleotides (4.5 nM) were mixed in annealing buffer (100 mM potassium acetate, 2 mM magnesium acetate, 30 mM HEPES pH 7.4), heated for 5 minutes at 95°C, and gradually cooled to room temperature. Annealed DNA fragments were ligated in *HpaI/SalI *sites of pShuttle-U6.

### Adenoviral vectors

Replication-deficient adenoviral vectors (E1/E3 deleted) Ad5.U6-HpTNFR1 and Ad5.U6-HpNS (hairpin non specific control, scrambled RNA from TNFR1) were prepared according to the AdEasy system [15], with the exception that replication-competent recombinant free viral particles were produced in E1 transformed N52E6 amniocyte cells [16]. Ad5.CMV-eGFP was a kind gift of Jay Kolls (Department of Pediatrics, Children's Hospital of Pittsburgh, PA, USA).

Viruses were purified by two consecutive CsCl_2 _gradient purifications and stored in small aliquots at -80°C in buffer containing 25 mM Tris, pH 8.0, 5 mM KCl, 0.2 mM MgCl_2_, 137 mM NaCl, 730 μM Na_2_HPO_4_, 0.1% ovalbumin, and 10% glycerol. The infectious particle titer (*ffu*) was determined by titrating vector stocks on 911 indicator cells and measuring viral capsid protein immunohistochemically 20 hours after transduction.

### Study design and histology

To study which organs are transduced after systemic or local treatment, Ad5.CMV-eGFP was injected into naïve DBA/1J mice intravenously or intra-articularly with 3 × 10^8 ^or 10^7 ^*ffu *adenovirus, respectively. One day later, liver, spleen, lung, knee joints, draining lymph nodes, blood, and bone marrow cells (BMCs) were isolated. They were fixated in 4% paraformaldehyde for 4 days for immunohistochemistry (IHC). After decalcification in 5% formic acid, specimens were processed for paraffin embedding. Tissue sections (7 μm) were stained with anti-GFP antibody. For mRNA measurement with reverse transcription-quantitative PCR (RT-qPCR), all parts were isolated.

DBA/1J mice were injected intravenously or intra-articularly 1 day after bCII booster (day 22) with 3 × 10^8 ^or 10^7 ^*ffu *adenovirus, respectively. For the siRNA (short interfering RNA) hairpin-treated mice, mice were sacrificed 3 days post-transduction, and synovium (i.a.), spleen, and liver (intravenous, i.v.) were isolated. Development of arthritis in front and hind paws was macroscopically monitored (scores between 0 and 2) until day 31. The macroscopic arthritis score is based on the clinical signs of inflammation in each paw and ankle; the maximum score is 8 (1 for each hind paw and 1 for the ankle). Mice were killed at day 26 or 31 by cervical dislocation. At day 26, synovial tissue explants (i.a.), spleen, and liver (i.v.) were removed. At day 31, ankle and knee joints (all groups) were removed and fixed in 4% paraformaldehyde for 4 days. After decalcification in 5% formic acid, specimens were processed for paraffin embedding. Tissue sections (7 μm) were stained with hematoxylin and eosin (cell influx) or safranin-*O *(cartilage proteoglycan depletion). Histological changes were scored in the patella/femur region on five semi-serial sections of the knee joint, spaced 70 μm apart. Scoring was performed by two observers without knowledge of the group, as described before. Histopathological changes were scored using the following parameters. Cartilage depletion, defined as the loss of proteoglycan content, was scored on a scale ranging from 0 to 3 per region, depending on the intensity of staining in the cartilage. Infiltration of cells was scored on a scale of 0 to 3 (0 = no cells, 1 = mild cellularity, 2 = moderate cellularity, 3 = maximal cellularity), depending on the number of inflammatory cells in the synovial cavity (exudate) or synovial tissue (infiltrate). Cartilage erosion was graded on a scale of 0 to 3, ranging from no damage to compete loss of articular cartilage.

### Immunohistochemistry

Paraffin sections were stained with rabbit anti-GFP (1:800) (#2555; Cell Signaling Technology, Danvers, MA, USA) overnight at 4°C. After washing, sections were incubated for 1 hour with biotinylated secondary antibody goat anti-rabbit-BIOT (1:400) (Vector Laboratories, Burlingame, CA, USA). After washing, sections were incubated for 30 minutes with Vectastain (1:400) (Vector Laboratories). Thereafter, sections were stained with 3,3'-diaminobenzidine and counterstained with hematoxylin, embedded in Permount (Thermo Fisher Scientific Inc., Rockford, IL, USA).

### Spleen cell isolation and antigen-presenting cell stimulation

Spleens were mashed and filtered, and erythrocytes were removed by osmotic shock. After washing, the splenic cell fraction was incubated in RPMI 1640 (Invitrogen Corporation, Carlsbad, CA, USA) at 37°C in 5% CO_2 _for 1 hour in order to separate adherent cells from nonadherent cells. The cells in the adherent cell fraction consisting mainly of macrophages are termed antigen-presenting cells (APCs). Splenic APCs were stimulated for 24 hours with 10 ng/mL TNFα (Abcam, Cambridge, UK). Cytokine production was analyzed using Luminex multianalyte technology. The Bioplex system in combination with multiplex cytokine kits (Bio-Rad, Veenendaal, the Netherlands) was used.

### Flow cytometry analysis

Total spleen cells obtained as described above were cultured (10^6^/mL) for 2 hours in RPMI 1640 (Invitrogen Corporation) supplemented with 10% FCS, penicillin-streptomycin, 1 mM pyruvate, 1 μl/mL Golgiplug inhibitor (BD Biosciences, San Jose, CA, USA), 10 ng/mL PMA (phorbol 12-myristate 13-acetate), and 1 μg/mL ionomycin. Thereafter, cells were labeled for 30 minutes at 4°C with antibody TCRβ-FITC (T-cell receptor beta-fluorescein isothiocyanate) (1:200) and CD4-APC (1:100) or their respective isotype control antibodies. Cells were washed and consecutively fixed and permeabilized using cytofix/cytoperm solution (BD Biosciences). Thereafter, cells were incubated with phycoerythrin (PE)-labeled antibodies interferon-gamma (IFNγ)-PE (1:200), interleukin-4 (IL-4)-PE (1:200), or IL-17-PE (1:500) (BioLegend, San Diego, CA, USA) or appropriate isotype controls for 30 minutes at 4°C in PBS containing 1% bovine serum albumin (BSA), 2% FCS, and 0.1% saponin. Analyses were performed on a BD FACSCalibur (BD Biosciences).

### Cytokine measurements

Synovial tissue explants were incubated for 1 hour at room temperature in 200 μL of RPMI 1640 supplemented with 0.1% BSA, penicillin-streptomycin, and 1% pyruvate. Subsequently, supernatant was harvested and centrifuged for 5 minutes at 1,000 *g*. Murine IL-1β, IL-6, and TNFα levels were determined using the Luminex multianalyte technology and the BioPlex system in combination with BioPlex Mouse Cytokine Assays (Bio-Rad Laboratories, Inc., Hercules, CA, USA). Cytokines were measured in 50 μL of washout medium. The sensitivities were 5, less than 3, and 5 pg/mL for IL-1β, TNFα, and IL-6, respectively.

### Luciferase measurements

NIH-3T3-5 × NF-κB-luciferase cells were seeded at 5 × 10^4 ^cells per well in a Krystal 2000 96-well plate (Thermo Labsystems, Brussels, Belgium). The day after, cells were transduced with adenovirus at the indicated multiplicity of infection (MOI) in 50 μL of DMEM for 4 hours at 37°C. Two days post-transduction, cells were stimulated with 10 ng/mL recombinant murine TNFα or IL-1β (R&D Systems, Abingdon, UK) for 6 hours and subsequently lysed in ice-cold lysis buffer (0.5% NP-40, 1 mM DTT, 1 mM EDTA, 5 mM MgCl_2_, 100 mM KCl, 10 mM Tris-HCl pH 7.5). Alternatively, TNFα was antagonized by preincubating cells for 1 hour with 10 μg/mL Enbrel (Wyeth Pharmaceuticals, Hoofddorp, The Netherlands). Luciferase activity was quantified using the Bright-Glo luciferase assay system (Promega Corporation, Madison, WI, USA) by adding an equal volume of Bright-Glo to the cell lysate. Luminescence was quantified in a luminometer (Lumistar; BMG Labtech GmbH, Offenburg, Germany), expressed as relative light units, and normalized to total protein content of the cell/tissue extracts using a BCA (bicinchoninic acid) protein assay kit (Thermo Fisher Scientific, Inc.).

### RNA isolation

Synovial and liver tissue was snap-frozen in liquid nitrogen and homogenized using a MagNa Lyser (Roche, Basel, Switzerland). Total RNA was extracted using TRI reagent (Sigma-Aldrich, St. Louis, MO, USA). Isolated RNA samples were treated with RNase-free DNase I (Qiagen, Venlo, The Netherlands) for 15 minutes. Synthesis of cDNA was accomplished by reverse transcription-PCR using an oligo(dT) primer and Moloney murine leukemia virus reverse transcriptase (Invitrogen Corporation).

### Quantitative polymerase chain reaction

qPCR was performed using SYBR Green PCR Master mix and the ABI 7000 Prism Sequence Detection system (Applied Biosystems Inc., Foster City, CA, USA) in accordance with the instructions of the manufacturer. Primers were designed over exon-exon junctions in Primer Express (Applied Biosystems Inc.) and used at 300 nM in the PCR (Supplementary methods in Additional file 1). PCR conditions were as follows: 2 minutes at 50°C and 10 minutes at 95°C, followed by 40 cycles of 15 seconds at 95°C and 1 minute at 60°C. Gene expression (cycle threshold, Ct) values were normalized using glyceraldehyde-3-phosphate dehydrogenase (*Gapdh*) as a reference gene (ΔCt = Ct_gene _- Ct_Gapdh_).

### Statistical analysis

Data are represented as mean ± standard error of the mean, and significant differences were calculated using Student *t *test, one-way analysis of variance, or Mann-Whitney *U *test, as indicated (GraphPad Prism; GraphPad Software, Inc., San Diego, CA, USA). *P *values of less than 0.05 were regarded as significant.

## Results

### Biodistribution after local and systemic administration of adenoviruses in mice

**Figure 1:**
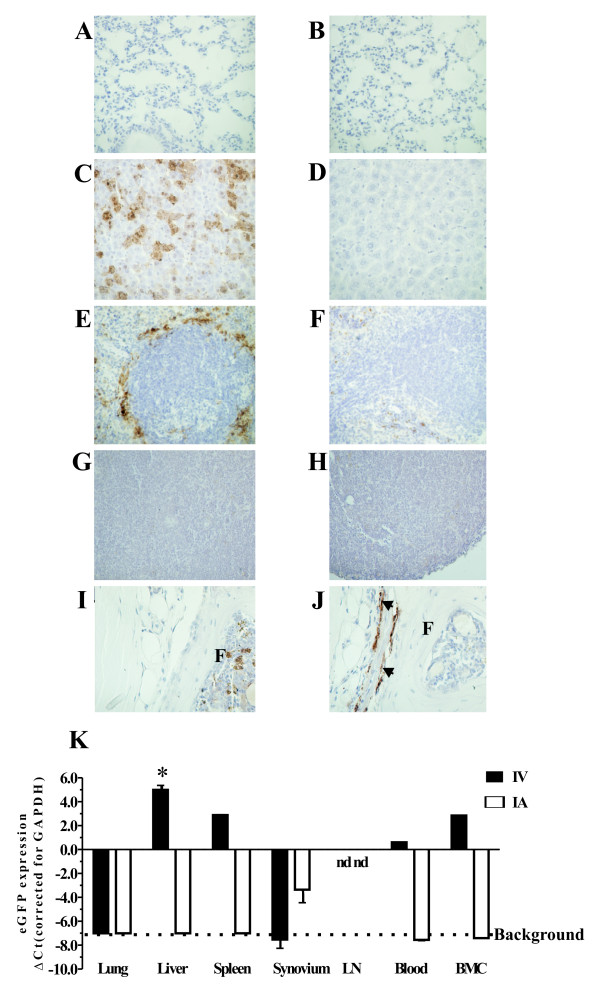
**Localization of transgene expression after local or systemic administration of adenoviral reporter vector in mice**. One day after collagen booster, nonarthritic DBA/1J mice were injected intra-articularly with 10^7 ^*ffu *or intravenously with 3 × 10^8 ^*ffu *Ad-eGFP. After administration, eGFP was assessed by immunohistochemistry in lung **(a, b)**, liver **(c, d)**, spleen **(e, f)**, lymph nodes (LNs) **(g, h)**, and synovium **(i, j) **after local (right frames) or systemic (left frames) treatment. Sites of eGFP-positive cells are indicated by arrows. **(k) **The expression of eGFP mRNA levels in each organ, blood, and bone marrow cells (BMCs). Draining LNs were negative on immunohistochemistry and quantitative polymerase chain reaction (not detected). Data are represented as the difference in cycle threshold (ΔCt) values compared with the housekeeping gene *GAPDH *(glyceraldehyde-3-phosphate dehydrogenase). mRNA levels that could not be detected are noted by 'nd' (not detectable). Background mean mRNA levels of eGFP are as low as the negative control. Bars represent mean ± standard error of the mean, and statistical differences were determined using Student *t *test. *P < 0.01. IA, intra-articular; IV, intravenous.

Ad5.CMV-eGFP was injected intravenously or intra-articularly 1 day after the bCII booster immunization in mice that had no clinical signs of arthritis. One day later, liver, spleen, lung, blood, BMCs, and synovium of the knee joints were isolated and prepared for IHC or processed for mRNA isolation. As expected, the systemically administered adenoviruses were scavenged by the RES primarily in liver and spleen. IHC detection of eGFP transgene expression, after systemic delivery of adenoviruses encoding for eGFP showed that in liver the Kupffer cells were predominantly transduced [17] and in the spleen the marginal metallophilic macrophages around the white pulpa [18] (Figure 1a, c, e, g, i). The synovium, draining lymph nodes, and lung remained negative on IHC. A more sensitive detection method is RT-qPCR, and at the mRNA level, the spleen, liver, but also blood and BMCs were positive for eGFP, whereas the synovium remained negative (Figure 1k). One day after i.a. injection, only SLCs, probably type B cells (based upon their morphology), were transduced as shown by RT-qPCR and IHC, whereas lung, liver, spleen, draining lymph nodes, blood, and BMCs were negative on IHC (Figure 1b, d, f, h, j).

### HpTNFR1 expression decreased TNFR1 mRNA expression and TNFα signaling *in vitro*

**Figure 2:**
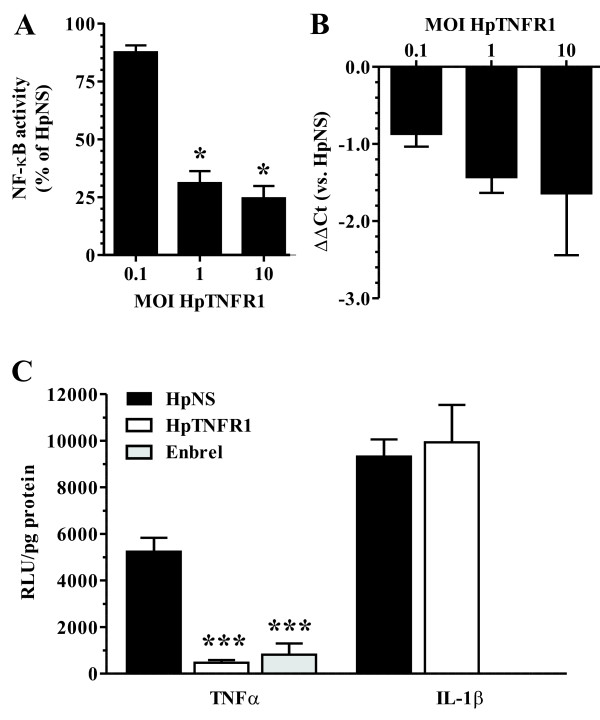
**Validation of hairpin construct targeting tumor necrosis factor receptor 1 (HpTNFR1) *in vitro***. **(a) **NIH-3T3-5 × NF-κB-luciferase cells were transduced at indicated multiplicity of infection (MOI) HpTNFR1 or hairpin non specific (HpNS) and, after 2 days, stimulated with 10 ng/mL mTNFα for 6 hours. Nuclear factor-kappa-B (NF-κB)-driven luciferase activity is represented as mean ± standard error of the mean (SEM) (n = 4) of percentages compared with the HpNS group. The numbers of HpNS transduced cells (doses MOI 10) with or without TNFα stimulation were 164,232 ± 864 and 21,555 ± 864 relative light units (RLU)/mg protein, respectively. **(b) **Expression of TNFR1 in NIH-3T3-5 × NF-κB-luciferase cells transduced at indicated MOI with HpTNFR1. Data are represented as the mean (n = 10) of the difference in TNFR1 ΔCt values compared with HpNS-treated group (ΔΔCt). **(c) **NIH-3T3-5 × NF-κB-luciferase cells were transduced at MOI 10 with HpTNFR1 or HpNS or left untreated. After 2 days, untreated cells were preincubated for 1 hour with 10 μg/mL Enbrel, and thereafter all groups were stimulated with 10 ng/mL mTNFα or mIL-β for 6 hours. Luciferase activity is represented as mean ± SEM (n = 4). Statistical differences were determined using analysis of variance with Bonferroni post-test. *P < 0.05; ***P < 0.001. Ct, cycle threshold; IL, interleukin; TNF, tumor necrosis factor.

RNAi-mediated downregulation of gene expression involves both translational repression and accelerated mRNA turnover [19]. To investigate the efficiency of TNFR1 gene silencing by shRNA expression, we transduced murine NF-κB-luciferase reporter fibroblasts with adenoviral vector encoding a hairpin construct targeting TNFR1 (HpTNFR1) or a scrambled control sequence (HpNS). After 2 days, cells were stimulated with TNFα for 6 hours, and TNFα-induced NF-κB activation and TNFR1 expression were quantified using a luciferase assay or qPCR, respectively (Figure 2a, b). At MOIs 1 and 10, we observed a strong reduction of NF-κB activation (70%) in the HpTNFR1-treated group as compared with the HpNS group. This was accompanied by two- and three-fold reductions (2^ΔΔCt^) of TNFR1 mRNA levels at MOIs 1 and 10, respectively. Next, we investigated the specificity of the TNFR1-targeting construct (Figure 2c). NF-κB-luciferase reporter fibroblasts were either transduced with HpTNFR1 or preincubated with a specific TNF antagonist (Enbrel) and then stimulated with TNFα or IL-1β. Both HpTNFR1 and Enbrel showed a strong reduction (90%) of TNFα-induced NF-κB activation. In contrast, HpTNFR1 treatment did not affect IL-1β-induced NF-κB activation, indicating the specific targeting of TNFR1-mediated signal transduction.

### TNFR1 silencing in synovial lining cells ameliorated arthritis

**Figure 3:**
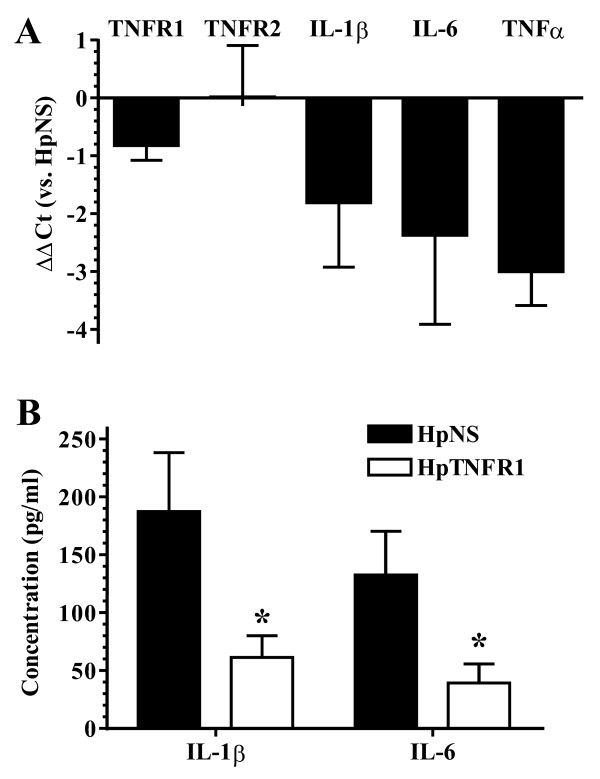
**Effects of silencing tumor necrosis factor receptor 1 (TNFR1) in synovial lining cells during streptococcal cell wall (SCW) arthritis**. Knee joints of naïve C57BL/6 mice were injected with 10^7 ^*ffu *hairpin construct targeting TNFR1 (HpTNFR1) or hairpin non specific (HpNS), and 2 days post-transduction, joints were challenged with 5 μg of SCW fragments. **(a) **Expression of indicated genes in synovial tissue at 24 hours after SCW challenge. Data are represented as mean ± standard error of the mean (SEM) (n = 3-6) of the difference in ΔCt values compared with the HpNS group (ΔΔCt). **(b) **Cytokine protein levels in 1-hour cultures of synovial tissue explants isolated at 24 hours after challenge. Bars represent mean ± SEM (n = 7), and statistical differences were determined using Student *t *test. *P < 0.05. Ct, cycle threshold; IL, interleukin; TNF, tumor necrosis factor.

**Figure 4:**
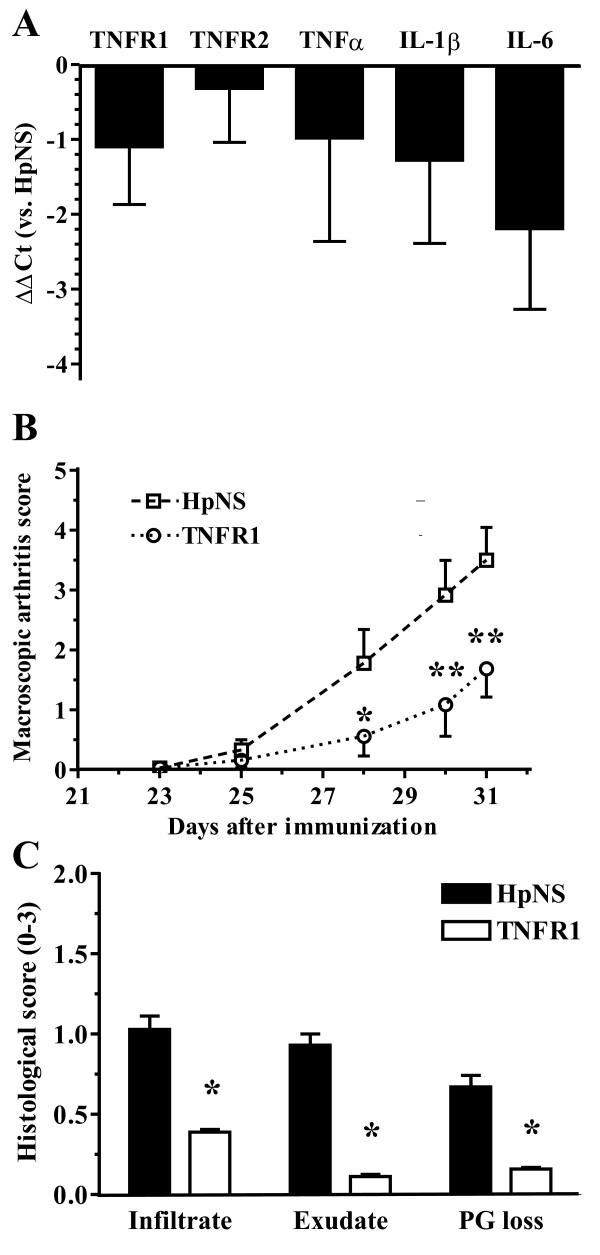
**Silencing of tumor necrosis factor receptor 1 (TNFR1) in synovial lining cells ameliorates collagen-induced arthritis (CIA)**. One day after collagen booster, knee joints of CIA-negative mice were injected with 10^7 ^*ffu *hairpin construct targeting TNFR1 (HpTNFR1) or hairpin non specific (HpNS). **(a) **Expression of indicated genes in synovial tissue at day 26 of CIA. Data are represented as mean ± standard error of the mean (SEM) (n = 6) of the difference in ΔCt values compared with the HpNS group (ΔΔCt). **(b) **Appearance of arthritis in fore and hind paws was monitored at indicated time points and scored for severity. **(c) **Histological analysis of inflammation ('infiltrate' and 'exudate') and proteoglycan depletion in patellar and femoral cartilage ('PG loss') from knee joints isolated at day 31. Data are represented as mean ± SEM (n = 9 mice), and statistical differences were calculated using Mann-Whitney *U *test. *P < 0.05, **P = 0.01. Ct, cycle threshold; IL, interleukin; TNF, tumor necrosis factor.

Previously, it was demonstrated that TNFR1 in SFs is essential to the development of strictly TNF-driven arthritis [10]. Therefore, we sought to investigate whether this mechanism also holds for arthritis models that are known to be partly TNF-dependent, including SCW [20] and CIA [1]. SLCs from knee joints of naïve C57BL/6 were transduced by i.a. injection with adenoviral vectors encoding HpTNFR1 or HpNS. One day thereafter, joints were challenged with 5-μg SCW fragments, and after 24 hours, synovial cytokine mRNA expression and protein levels were measured by qPCR and Luminex, respectively (Figure 3a, b). TNFR1, but not TNFR2, mRNA level was decreased (twofold) in synovial tissue explants from the HpTNFR1-treated group. In addition, we observed a strong reduction (more than threefold) in mRNA levels of IL-1β, IL-6, and TNFα. Corresponding with these results, protein levels of IL-1β and IL-6 were significantly reduced in the HpTNFR1 group compared with the HpNS group. Next, knee joints of CIA-negative mice were transduced with HpTNFR1 or HpNS at day 1 after booster (day 22). RT-qPCR analysis at day 26 showed a strong (more than fourfold) reduction in synovial mRNA levels of IL-1β, IL-6, and TNFα (Figure 4a). Arthritis development was monitored until day 31 (Figure 4b). While CIA incidence was equal between treatments, TNFR1 silencing clearly reduced macroscopic arthritis severity. Histology taken at day 31 revealed protection against cartilage destruction and a significant reduction in the amount of synovial inflammatory cell infiltrate and joint space inflammatory cell exudate (Figure 4c).

### TNFR1 silencing in the reticuloendothelial system prevented collagen-induced arthritis development

**Figure 5:**
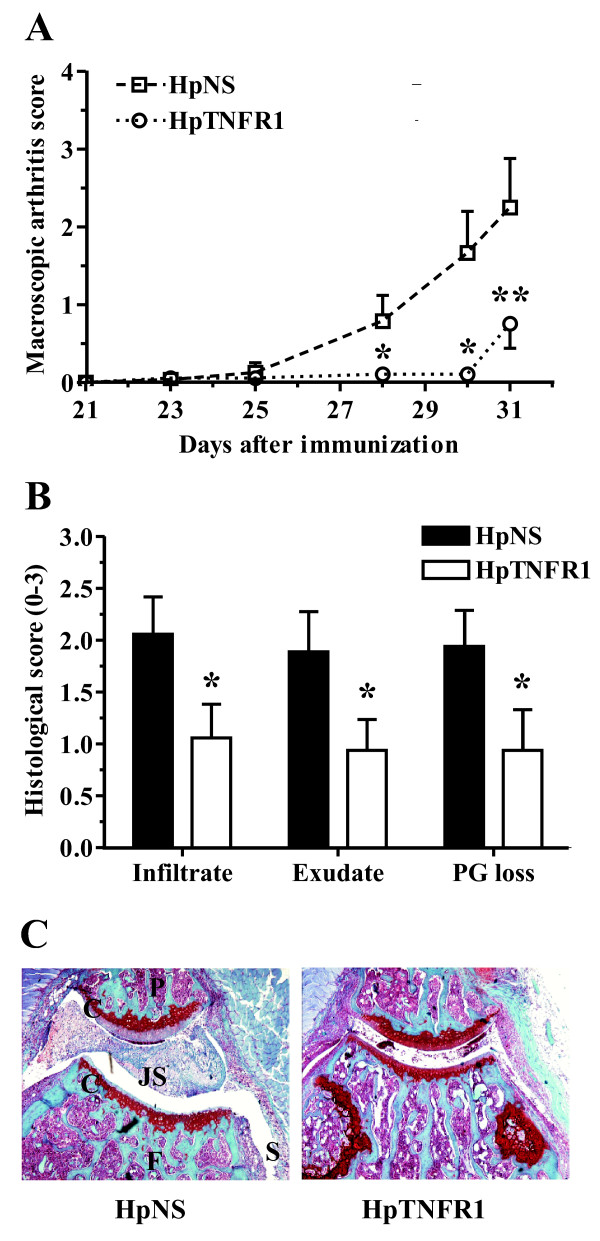
**Tumor necrosis factor receptor 1 (TNFR1) silencing in the hepatic and splenic reticuloendothelial system ameliorates collagen-induced arthritis (CIA)**. One day after collagen booster, CIA-negative mice were injected intravenously with 3 × 10^8 ^*ffu *hairpin construct targeting TNFR1 (HpTNFR1) or hairpin non specific (HpNS). **(a) **Appearance of arthritis in fore and hind paws was monitored at indicated time points and scored for severity. **(b) **Histological analysis of inflammation ('infiltrate' and 'exudate') and proteoglycan depletion in patellar and femoral cartilage ('PG loss') from knee joints isolated at day 31. Data are represented as mean ± standard error of the mean (n = 6 mice), and statistical differences were calculated using Mann-Whitney *U *test. *P < 0.05, **P < 0.005. **(c) **Representative picture of safranin-*O*-stained tissue sections of knee joints from mice treated systemically with HpNS or HpTNFR1. Original magnification × 40. C, cartilage; F, femur; JS, joint space; P, patella; S, synovium.

Recently, it was shown that TNFR1 silencing in the radiosensitive hematopoietic compartment aggravates disease in CIA [12]. Secondary lymphoid organs, such as liver and spleen, are rich in mature and functional cells of hematopoietic origin, such as lymphocytes, monocytes, and APCs. To delineate the function of TNFR1 in hepatic and splenic cells during arthritis, CIA-negative mice (collagen type II immunized mice without macroscopic signs of arthritis) were injected intravenously with HpTNFR1 or HpNS at day 1 after booster injection (day 22). We monitored arthritis development until day 31 (Figure 5a). Up to day 30, the incidence of arthritis in the paws of mice treated with HpTNFR1 (40%) was considerably reduced compared with HpNS treatment (83%) (data not shown). In addition, macroscopic arthritis scores were significantly reduced in the TNFR1 group. Histology of knee joints taken on day 31 confirmed a significant reduction in joint inflammation and revealed a strong suppression of cartilage proteoglycan depletion (Figure 5b, c).

### TNFR1 silencing in antigen-presenting cells reduced the number of T helper cells in spleen and dampened the acute-phase response in liver

**Figure 6:**
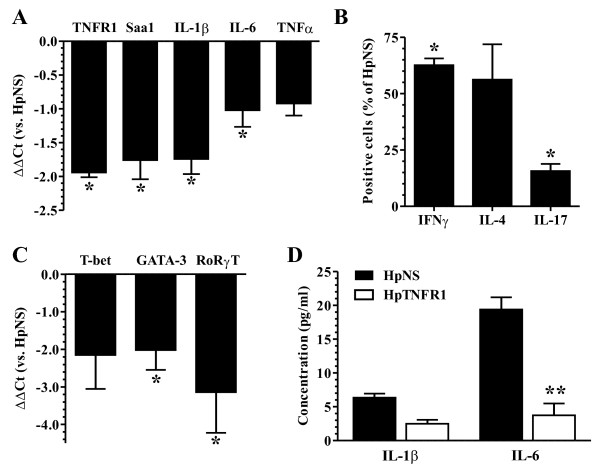
**Effects of tumor necrosis factor receptor 1 (TNFR1) silencing in the hepatic and splenic reticuloendothelial system**. One day after collagen booster, mice negative for collagen-induced arthritis were injected intravenously with 3 × 10^8 ^*ffu *hairpin construct targeting TNFR1 (HpTNFR1) or hairpin non specific (HpNS). **(a) **Expression of indicated genes in liver isolated at day 26. Data are represented as mean (n = 5) of the difference in ΔCt values compared with the HpNS group (ΔΔCt). **(b) **Analysis of intra-cellular cytokine expression in T cells isolated from spleen at day 26. Data are represented as mean ± standard error of the mean (SEM) (n = 4) of the percentage of positive cells compared with the HpNS group. **(c) **Expression of indicated genes in isolated splenic T cells. Data are represented as mean (n = 5) of the difference in ΔCt values compared with the HpNS group (ΔΔCt). **(d) **Secreted cytokine levels from splenic antigen-presenting cells stimulated for 24 hours with 10 ng/mL mTNFα. Data are represented as mean ± SEM (n = 5). Statistical differences were calculated using analysis of variance with Bonferroni post-test. *P < 0.05, **P < 0.01. Ct, cycle threshold; IL, interleukin; TNF, tumor necrosis factor.

To elaborate on the mechanisms behind HpTNFR1-mediated prevention of CIA, we analyzed proinflammatory gene expression in liver at disease endpoint by qPCR (Figure 6a). This showed a significantly reduced (>3-fold) expression of TNFR1, IL-1β, IL-6 and the acute phase gene Saa1. To study the effects of TNFR1 silencing in spleen, we performed FACS and qPCR analyzes on the splenocytes (Figure 6b, c) and cytokine measurements on the APC fraction (Figure 6d). FACS (fluorescence-activated cell sorting) analysis showed a significant reduction in the number of CD4^+^/TCRβ T cells, stained intracellularly for T helper 1 (Th1) (IFNγ), Th2 (IL-4), or Th17 (IL-17) cytokine expression. This was accompanied by a strong decrease (more than fourfold) in mRNA expression of their respective transcription factors (T-bet, GATA-3, and RoRγT). Since both IL-1 and IL-6 have been described as crucial cytokines in T-cell expansion and differentiation [21,22], we measured their production by TNFα-stimulated APCs in HpTNFR1- and HpNS-treated groups. Indeed, secreted IL-6 protein levels were significantly reduced in HpTNFR1-treated as compared with HpNS-treated groups. Together, these data demonstrate a clear proinflammatory role of TNFR1 in SFs and splenic APCs.

## Discussion

The pleiotropic biological and immunological activities of TNFα are determined by its cellular localization (transmembrane or soluble) [23-26] and the cell-specific relative abundance of its respective receptors, TNFR1 and TNFR2 [9,12,27,28]. The role of TNF as pivotal mediator of the cytokine cascade in inflammation and RA pathogenesis has been unequivocally established, but the relative contributions of specific cell types and TNF receptors have not been fully elucidated. Delineating the role of TNF and its receptors in different tissues and cell types relevant to disease may contribute to a better and safer TNF-targeting strategy in RA patients. While a number of studies using TNFR1-deficient mice have established the global contribution of signal transduction through this receptor in CIA [8,9,11,29], cell-specific functions of TNFR1 have thus far been studied only in SFs, bone marrow-derived macrophages, and radiosensitive hematopoietic cells [10,12,30,31]. In this study, we have demonstrated that, after local treatment in the knee joint, only the SLCs were transduced and that there was no spillover to other organs. Gouze and colleagues [32] have shown that, after i.a. adenovirus delivery, 75% to 90% of the transduced cells are positive for fibroblast markers (CD90, CD29, and VCAM-1) and no transduced cells were positive for the macrophage marker CD11b. Ten percent of the transduced cells are positive for the APC marker CD86. After systemic delivery, predominantly liver and spleen were transduced, while synovium remained negative. It is well documented that systemic i.v. delivery of adenoviruses targets the Kupffer cells in the liver [33] and marginal zone macrophages in spleen [34]. Stone and colleagues [35] demonstrated that adenoviruses in the circulation become opsonized by blood platelets and that these aggregates are sequestered in the RES. Interestingly, depletion of synovial tissue macrophages [36] or the macrophages in spleen and liver [37] after local or systemic administration of clodronate-encapsulated liposomes demonstrates the crucial role of both the local and systemic macrophages in mediating experimental arthritis. For this, we can conclude that TNFR1-mediated signaling in joint, liver, and spleen RES compartments contributes to the local joint inflammation and the development of autoimmunity during experimental arthritis.

The contribution of TNFR1-mediated signaling in SLCs to joint inflammation was investigated after SCW challenge. In the acute phase, SCW arthritis represents an innate immune response against SCW fragments that is driven by direct activation of macrophages [38,39]. TNFR1 silencing in SLCs resulted in a significant reduction of secreted IL-6 and IL-1β levels in the joint, which indicates an inhibition of the local cytokine cascade. The reduction of IL-6 is most likely a direct effect of TNFR1 silencing in SLCs since previous studies demonstrated that TNF-induced IL-6 secretion in human RA SFs is mediated exclusively through TNFR1 [40,41]. In contrast, hematopoietic cells (neutrophils and monocytes), but not mesenchymal cells (SFs), were identified as the main source of IL-1 in TNF-driven joint pathology [42]. The observed reduction of IL-1β suggests that TNF signaling in SLCs plays an important role in chemoattraction of inflammatory cells. Indeed, histological analysis of HpTNFR1-treated joints in CIA showed almost complete prevention of IL-1-induced cartilage proteoglycan loss, which was accompanied by an impressive reduction of inflammatory cell influx. We have revealed, in line with the study of Armaka and colleagues [10], a dominant role of TNFR1-mediated signaling in SLCs in joint inflammation.

Remarkably, we found that TNFR1 silencing in knee joints also protected the ipsilateral ankle joints in CIA mice. While such distal effects have been described before in local gene therapy approaches [43-45], the underlying mechanism is still not fully understood. However, such an effect might point toward a role of local TNFR1-mediated signaling in the development of autoimmunity. In support of this, previous investigations using periarticular delivery of secreted transgenes, vIL-10 and TNFR, in CIA showed that distant anti-arthritic effects coincided with a reduction of specific collagen antibody titers and modulation of T-cell responses, respectively [45-47]. Notably, the beneficial systemic effects of periarticular TNFR gene therapy correlated well with circulating levels of the transgene [45]. In the absence of transgene spillover to the circulation, distal effects have been attributed to antigen-primed APCs exposed to the therapeutic transgene traveling from treated to untreated joints [47-49]. In our approach, TNFR1 silencing was restricted to SLCs that would exclude transgene spillover or direct modulation of APCs as a causative for systemic effects. However, local TNFR1 treatment reduced local levels of IL-6 and IL-1β. Both cytokines are implicated in APC function, which is in turn a prerequisite for induction of auto-reactive CD4^+ ^T cells and autoimmunity. Eriksson and colleagues [50] demonstrated that IL-1 receptor type I is required for efficient activation of dendritic cells (DCs). IL-6 switches the differentiation of monocyte-derived APCs from DCs to macrophages [51]. The observed reduction of both IL-1β and IL-6 synthesis in the inflamed joint may result in the development of immature DCs, a differentiation state associated with a tolerogenic function of these cells. Alternatively, tolerogenic DCs can be induced by IL-10, a cytokine that inhibits the synthesis of IL-1 and IL-6 in monocytes and other cell types [52]. Alternatively, TNFR1 treatment might have affected the APC-like function of SLCs. Although SFs are not considered to be professional APCs, approximately 60% to 70% of SFs in the rheumatic joint express MHC (major histocompatibility complex) class II molecules and have the capacity to serve as accessory cells for superantigen-mediated T-cell activation [53-55]. Importantly, the interaction between cytokine-activated T cells and SFs was found to be dependent on transmembrane TNFα on the surface of T cells and resulted in increased production of IL-6 and chemokine IL-8 [56]. Indeed, we found strongly decreased IL-6 production in HpTNFR1-treated joints, which may abrogate the ability of SLCs to present auto-antigens found within joint tissues.

Strikingly, systemic treatment with HpTNFR1 ameliorated CIA almost to the same extent as local treatment. We have previously shown that SOCS3 (suppressor of cytokine signaling-3) overexpression in splenic APCs ameliorates CIA via a general suppression of Th subsets [18]. Similarly, we observed a reduction in the number of Th1 (IFNγ, T-bet), Th2 (IL-4, GATA-3), and Th17 (IL-17, RoRγT) cells upon TNFR1 in splenic APCs after antigen booster injection. In line with these similar findings, it has been demonstrated that TNFR1-deficient murine myocardiocytes show increased expression of SOCS3 and reduced IL-6 secretion upon TNF infusion [57]. We have confirmed that the splenic APCs from HpTNFR1-treated mice produce markedly less IL-6 and IL-1β after TNF stimulation. As these cytokines are crucially involved in Th17 differentiation [21,22], the observed large reduction of Th17 numbers in spleen is not unexpected. Thus, TNFR1 modulation in the RES has a clear-cut effect on immunity in CIA.

In a side-by-side comparison, we have demonstrated equal efficacies of local and systemic RNAi-mediated TNFR1-targeting gene therapy in alleviating CIA. Importantly, cell-specific gene therapeutic targeting of TNFR1 clearly modulated proinflammatory effects of TNFα without interfering with protective effects of TNF signaling that have been described in hematopoietic cells [11,12]. It will be interesting to investigate whether local or systemic TNFR1 knockdown gives a different outcome in CIA when using a therapeutic regimen.

## Conclusions

Specific silencing of TNFR1 in SLCs, hepatic and splenic RES by respectively local or systemic delivery of Ad5 virus encoding for small hairpin RNA against TNFR1 revealed a dominant and clear proinflammatory role of TNF signaling in these cells during CIA. Systemic treatment dampened the liver acute-phase response and reduced proliferation of Th subsets in spleen. Local treatment inhibited the proinflammatory cytokine cascade in the joint. Gene therapeutic targeting of TNFR1 may be a promising and safer approach for TNFα blockade in RA patients.

## Abbreviations

APC: antigen-presenting cell; bCII: bovine collagen type II; BMC: bone marrow cell; BSA: bovine serum albumin; CIA: collagen-induced arthritis; Ct: cycle threshold; DC: dendritic cell; DMEM: Dulbecco's modified Eagle's medium; FCS: fetal calf serum; Gapdh: glyceraldehyde-3-phosphate dehydrogenase; HpNS: hairpin non specific; HpTNFR1: hairpin construct targeting tumor necrosis factor receptor 1; i.a.: intra-articular; IFNγ: interferon-gamma; IHC: immunohistochemistry; IL: interleukin; i.v.: intravenous; MOI: multiplicity of infection; NF-κB: nuclear factor-kappa-B; PBS: phosphate-buffered saline; PCR: polymerase chain reaction; PE: phycoerythrin; qPCR: quantitative polymerase chain reaction; RA: rheumatoid arthritis; RES: reticuloendothelial system; RNAi: RNA interference; RT-qPCR: reverse transcription-quantitative polymerase chain reaction; SCW: streptococcal cell wall; SF: synovial fibroblast; shRNA: short hairpin RNA; SLC: synovial lining cell; SOCS3: suppressor of cytokine signaling-3; TCRβ: T-cell receptor beta; Th: T helper; TNFα: tumor necrosis factor-alpha; TNFR: tumor necrosis factor receptor.

## Competing interests

The authors declare that they have no competing interests.

## Authors' contributions

OJA helped to acquire data and contributed to the study design, statistical and data analysis, interpretation of data, and drafting of the manuscript. SV, BTvdB, and MBB helped to acquire data. JG, SA-R, and FAvdL contributed to the study design, statistical and data analysis, interpretation of data, and drafting of the manuscript. WBvdB conceived of the study and helped draft the manuscript. All authors read and approved the final manuscript.

## Supplementary Material

Additional file 1 **Supplemental Methods**. Primerdesign. Click here for file
