# Supplementary material for: A crucial role for tumor necrosis factor receptor 1 in synovial lining cells and the reticuloendothelial system in mediating experimental arthritis
Source: Arthritis Res Ther. 2010 Apr 6;12(2):R61. doi: 10.1186/ar2974 (PMC2888212; doi:10.1186/ar2974)
Supplement: Additional file 1 — Supplemental Methods. Primerdesign. [file ar2974-S1.PDF]

Gene	Gene number	FW primer 5'→ 3'	RV primer 5'→ 3
**TNFRI**	**[EMBL:L26349]**	cctacctcctccgcttgca	acagcaccgcagtacctgagt
**TNFR2**	**[EMBL:M59377]**	ccaaactccaagcatccttacatc	cactccaacaatcagaccaattg
**IL-1β**	**[EMBL:M15131]**	ggacagaatatcaaccaacaagtgata	gtgtgccgtctttcattacacag
**TNFα**	**[EMBL:D84199]**	cagaccctcacactcagatcatct	cctccacttggtggtttgcta
**IL-6**	**[EMBL:BC132458]**	caagtcggaggcttaattacacatg	attgccattgcacaactcttttct
**T-bet**	**[EMBL:AF093099]**	caacaaccccctttgccaaag	tcccccaagcagttgacagt
**GATA-3**	**[EMBL:X55123]**	agaaccggcccсttatcaa	agttcgcgcaggatgtcc
**RoRγT**	**[EMBL:AJ132394]**	ctgtcctgggctaccctactga	aagggatcacttcaatttgtgttctc
**Saa-1**	**[EMBL:L22190]**	tgctgagaaaatcagtgatggaa	ggtcagcaatggtgtcctcat
**Gapdh**	**[EMBL:GU214026]**	ggcaaattcaacggcaca	gttagtggggtctcgctcctg
